# Spontaneous tibiotalar arthrodesis as a complication of acute tibial osteomyelitis due to Panton-Valentine leukocidin-producing *Staphylococcus aureus*: a case report

**DOI:** 10.1186/1752-1947-6-202

**Published:** 2012-07-17

**Authors:** Dimitri Ceroni, Rebecca Anderson de la Llana, Tristan Zand, Léopold Lamah, Denis Dominguez, Geraldo De Coulon, Victor Dubois-Ferrière

**Affiliations:** 1 Pediatric Orthopedic Unit, University Hospital of Geneva, Geneva, 14 CH-1211, Switzerland; 2 Department of Child and Adolescent, Service of Pediatric Orthopedics, University Hospitals of Geneva, 6, rue Willy Donzé, Geneva, 14 1211, Switzerland; 3 Department of Radiology, University of Geneva Hospitals (HUG), Geneva, 14 CH-1211, Switzerland

## Abstract

**Introduction:**

Strains of Panton-Valentine leukocidin-producing *Staphylococcus aureus* producing a new pattern of disease have emerged worldwide. Infection with these bacteria typically presents as a life-threatening infection of soft tissues and bones, and may cause potentially devastating consequences.

**Case presentation:**

We report a case of osteoarticular infection caused by Panton-Valentine leukocidin-producing *Staphylococcus aureus*. A 12-year-old Caucasian girl presented with acute osteomyelitis of the tibia associated with toxic shock syndrome, which was complicated by an unexpected spontaneous ankle arthrodesis.

**Conclusions:**

Osteoarticular infections due to Panton-Valentine leukocidin-producing *Staphylococcus aureus* appear to be severe, and are characterized by their tendency to evolve towards serious complications. This case highlights the need for early and aggressive surgical procedures in conjunction with appropriate antimicrobial therapy and regular long-term follow-up.

## Introduction

Panton-Valentine leukocidin (PVL) is a pore-forming toxin [1], secreted by 2% to 5% of *Staphylococcus aureus* strains. This exotoxin induces polymorphonuclear cell death via necrosis or apoptosis, and the subsequent release of leukocytic cytokines [2]. Induction of leukocyte apoptosis by *S. aureus* may cause extensive tissue necrosis and compromise the antimicrobial response, thereby facilitating bacterial spread [2]. The presence of PVL genes is associated with an increased risk of more severe systemic inflammation and serious infection requiring intensive care [3,4]. PVL-positive staphylococcal infections may lead to life-threatening infections of soft tissues, such as necrotizing hemorrhagic pneumonia, associated with a high mortality rate [5-7]. Bone and joint infections with PVL-producing *S. aureus* (PVL + SA) are more aggressive, present a different evolution, and have a probably higher rate of orthopedic complications [8]. The purpose of this paper is to report an unusual and severe complication resulting from an osteoarticular infection of PVL + SA.

## Case presentation

A previously healthy 12-year-old Caucasian girl was brought to the emergency department of our hospital complaining of a swollen left ankle and fever. She had a history of ankle trauma three days before. Her vital signs included a temperature of 40°C, a pulse rate of 132 beats/minute, a respiratory rate of 22 breaths/minute and blood pressure of 90/55 mmHg. Her left leg and ankle were diffusely tender. There was redness on her foot and the medial aspect of her leg. Laboratory results showed a white blood cell count of 21,000 cells/mm^3^, an elevated C-reactive protein (200 mg/dL; reference range: 0 to 10 mg/dL) and an abnormal erythrocyte sedimentation rate (78 mm/h).

**Figure 1:**
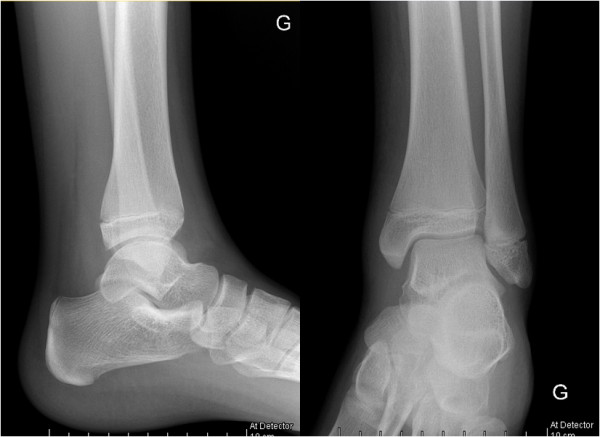
**Initial conventional X-ray of the affected tibia.** Early frontal and lateral conventional X-rays only reveal discrete peritibial soft tissue infiltration.

**Figure 2:**
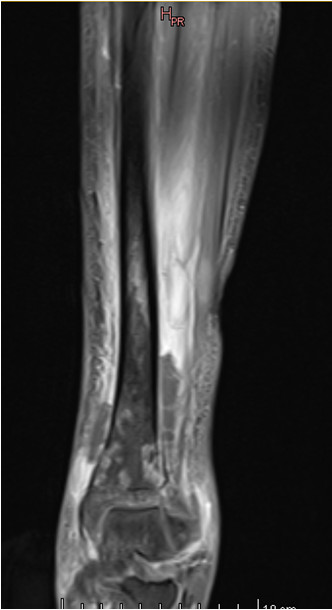
**Initial magnetic resonance images of the affected tibia.** Overall extension is best understood on magnetic resonance images with extensive heterogeneous medullar and pericortical enhancement on T1-weighted fat-suppressed coronal images.

**Figure 3:**
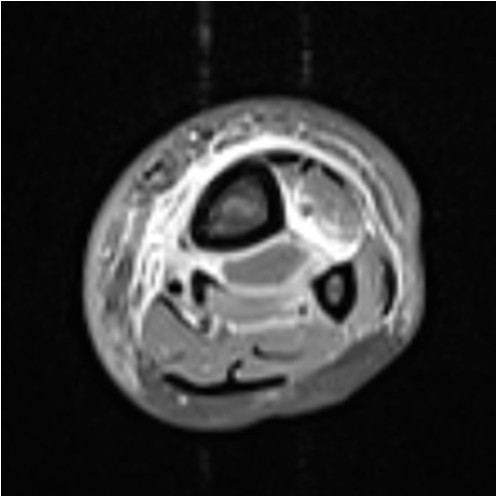
**Initial magnetic resonance images of the affected tibia.** The peripheral reactive effusion is explicit on liquid sensitive inversion-recovery T2-weighted axial images.

**Figure 4:**
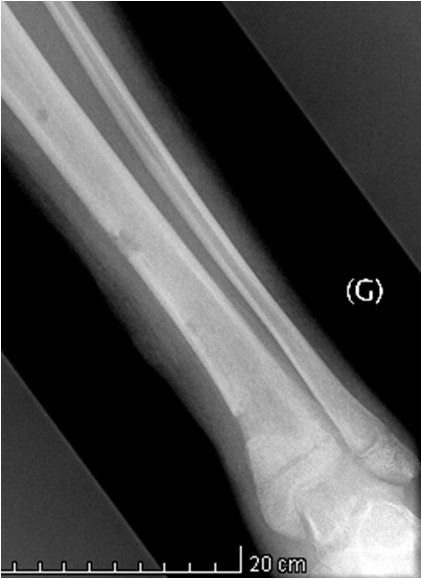
**Conventional X-ray of the affected tibia 42 days after surgical drainage and the onset of antibiotic treatment.** Frontal X-ray control after pharmacological and surgical treatment illustrates the diffuse bone remodeling and sclerosis. Major narrowing of the tibiotalar joint space illustrates the aggressiveness of the overall infectious process.

**Figure 5:**
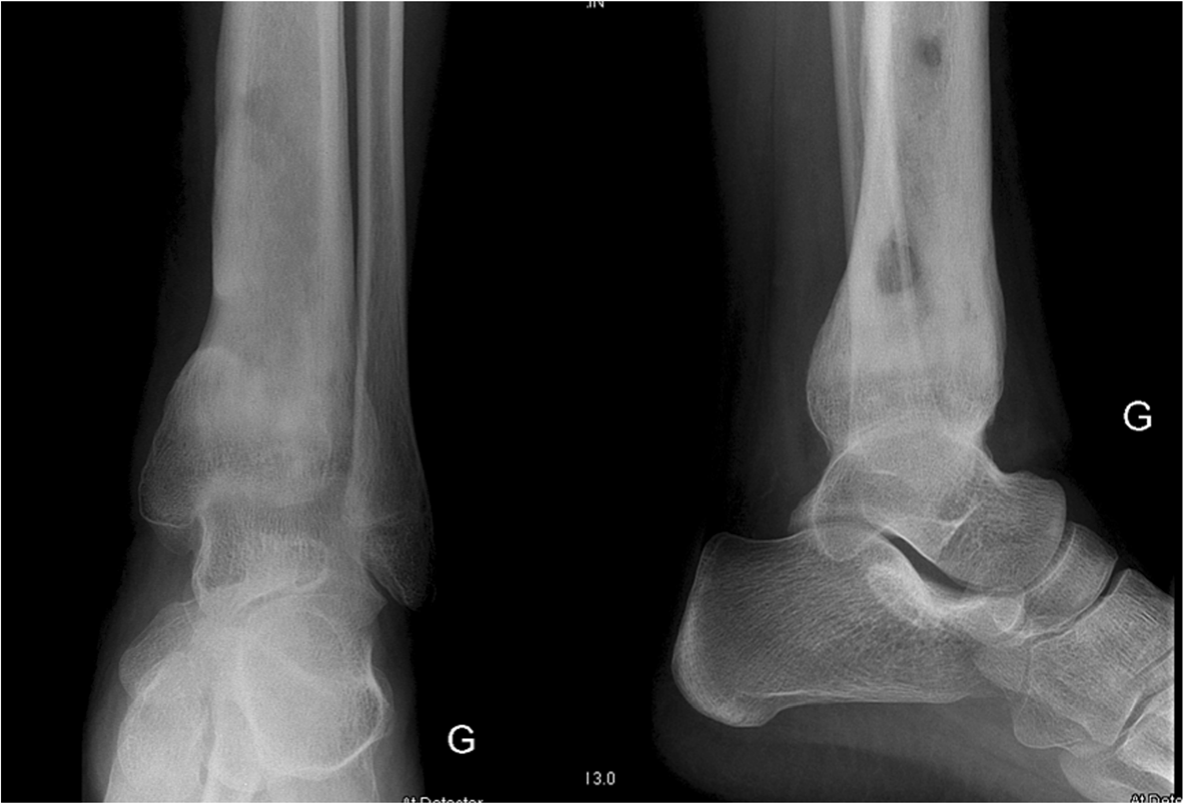
**Late control X-rays of the affected tibia three and a half months after disease onset.** Extensive bone remodeling consisted of cortical thickening, diffuse sclerosis, anatomical deformation and tibiotalar fusion.

Initial conventional X-rays were normal (Figure 1), but magnetic resonance imaging (MRI) demonstrated a large osteomyelitis of her left tibia with an increased signal intensity in its bone marrow, spreading up to her mid-tibia (Figures 2 and 3). The MRI of the tibia showed the presence of intramedullary abscesses and significant edema of the surrounding muscles (Figure 3). Our patient was taken to the operating theater, where she underwent multilevel surgery with pus drainage and extensive cleaning of the intramedullary cavity, using 12 liters of saline solution. An intravenous empiric antibiotic therapy was immediately started with flucloxacillin and gentamicin. Abscess cultures were positive for methicillin-sensitive *S. aureus*, and a polymerase chain reaction (PCR) was positive for PVL. Because our patient remained symptomatic, she had revision surgery under general anesthesia four times over a 17-day period. New recurrent abscesses were drained each time, and the tibial diaphysis was thoroughly washed using 12 liters of isotonic saline solution. After the fifth intervention, we finally obtained a resolution of local and biological signs of infection. Intravenous antibiotics were given for four weeks, followed by an oral antibiotic for another four weeks. Six weeks after the onset of the infection, we noted a severe restriction of the range of motion of her left ankle, and an X-ray showed an alteration of the tibiotalar joint line (Figure 4). Two months later, she had total fusion of the ankle, fortunately in a functional position (Figure 5).

## Discussion

PVL is an exotoxin that induces cytotoxic and cytolytic changes in leukocytes. Complications of infections with PVL + SA include septic thrombosis, purpura fulminans and a redoubtable necrotizing pneumonia [9]. Clinical and biological signs of pulmonary disease are characterized by a combination of hemoptysis, multilobar alveolar infiltrations and leukopenia [9]. Severe pulmonary infection with PVL + SA has a mortality rate up to 75% despite intensive medical care [10]. In those cases, intensive care, antibiotics, aggressive surgical treatment and the additional application of immunoglobulins is the recommended treatment [9]. It is nevertheless important to keep in mind that the onset of necrotizing pneumonia may happen during the evolution of osteoarticular infection.

Interest in PVL + SA has progressively increased over the last years, probably due to the continuing improvement of PVL + SA detection methods with novel PCR assays. However, to date, only a few papers have focused specifically on the aspect of bone and joint infections [7-9,11-13]. The true incidence of osteoarticular infections caused by PVL + SA is unknown, probably because the number of infections due to this strain is underestimated. Ellington *et al*. found that only 1.6% of *S. aureus* from patients with bacteremia in the UK were PVL-positive [12]. In the USA, the PVL-positive methicillin-resistant *S. aureus* (MRSA) strain is responsible for 20% of bloodstream infections [8], and in Germany, PVL + SA became the second most frequent MRSA isolate [14]. In our department, we noted only two cases of osteoarticular infections due to PVL + SA.

From an orthopedic point of view, the emergence of osteoarticular infections due to PVL + SA has complicated the conventional management of these infections in children [15]. PVL + SA infections of bone and joints induce more often a concomittant myositis or pyomyositis than PVL negative SA infections [3]. Furthermore, the bone infections are more severe, presenting extended diaphysitis and bone abscesses, and causing more complications [8].

Therefore, osteoarticular infections due to such strains need longer antibiotic courses and various surgical procedures [8,15]. In children, orthopedic consequences may arise if the growth cartilage is involved, or if the articular cartilage is damaged by the infection; unfortunately the early diagnosis of these sequelae is often difficult.

In our case, our patient sustained an unusual complication because joint arthrodesis is not commonly encountered after PVL + SA infections. Nevertheless, the biological characteristics of PVL + SA may explain, from a theoretical point of view, the onset of spontaneous arthrodesis after an osteoarticular infection. In fact, PVL specifically interacts with and lyses polymorphonuclear leukocytes, and thus contributes to the inhibition of infection clearance by the host immune system, thereby enabling staphylococcal species to persist [16]. If the infection is not quickly cleared by the host, the potent activation of the immune response, in association with high levels of cytokines and reactive oxygen species, increase the release of host matrix metalloproteinases and other collagen-degrading enzymes, which in conjunction with bacterial toxins lead to joint destruction [17]. The polymorphonuclear response with subsequent release of these proteolytic enzymes can lead to permanent destruction of the intra-articular cartilage and subchondral bone loss in as little as three days [17]. In addition, metalloproteinases and the antigen-induced inflammatory response may persist and continue to damage the joint architecture even after the infection has been cleared [18-20].

## Conclusions

PVL + SA seems to be an emerging and dangerous pathogen of osteoarticular infections in children. Its evolution is more aggressive, treatment more difficult, and complications more severe. Even if infections caused by PVL + SA are currently still uncommon, a PCR-based assay for toxin gene profiling should be performed in cases of severe and extended osteoarticular infections or when severe sepsis occurs. Orthopedic surgeons must be aware of the possible future changes in the evolution and behavior of osteoarticular infections in children. There is probably a subtle balance between an effective immune response, which eliminates the infecting organism from the host, and the overactivation of this response that causes the majority of infection-related joint destruction. For this reason, it is now unanimously recognized that a surgical approach is mandatory for the management of PVL + SA osteoarticular infections, in conjunction with an appropriate antimicrobial therapy, and long-term follow-up of the affected children is necessary to recognize late-onset sequelae.

## Consent

Written informed consent was obtained from the patient’s legal guardian for publication of this manuscript and accompanying images. A copy of the written consent is available for review by the Editor-in-Chief of this journal.

## Competing interests

The authors declare that they have no competing interests.

## Authors’ contributions

CD was the major contributor in writing the manuscript. AR drafted and critically revised the manuscript. TZ realized the interpretation of the radiological materials. LL drafted and critically revised the manuscript. DD operated on our patient and critically revised the manuscript. DCG operated on our patient and critically revised the manuscript. DFV contributed toward the study by preparing the photographs, by revising the manuscript and by performing the editorial work. All authors read and approved the final manuscript.
